# Associations of genome-wide and regional autozygosity with 96 complex traits in old order Amish

**DOI:** 10.1186/s12864-023-09208-5

**Published:** 2023-03-20

**Authors:** Megan T. Lynch, Kristin A. Maloney, Huichun Xu, James A. Perry, Regeneron Genetics Center, Alan R. Shuldiner, Braxton D. Mitchell

**Affiliations:** 1 Department of Medicine Baltimore, University of Maryland School of Medicine, Maryland, MD USA; 2 University of Maryland School of Medicine, Program for Personalized and Genomic Medicine, Baltimore, MD USA; 3 Baltimore Veterans Administration Medical Center Geriatrics Research and Education Clinical Center, Baltimore, MD USA; 4 Regeneron Genetics Center LLC, Tarrytown, NY USA

**Keywords:** Autozygosity, Runs of Homozygosity, Founder population genetics, Amish

## Abstract

**Background:** Autozygosity, the proportion of the genome that is homozygous by descent, has been associated with variation in multiple health-related traits impacting evolutionary fitness. Autozygosity (FROH) is typically measured from runs of homozygosity (ROHs) that arise when identical-by-descent (IBD) haplotypes are inherited from each parent. Population isolates with a small set of common founders have elevated autozygosity relative to outbred populations.** Methods:** In this study, we examined whether degree of autozygosity was associated with variation in 96 cardiometabolic traits among 7221 Old Order Amish individuals residing in Lancaster County, PA. We estimated the average length of an ROH segment to be 6350 KB, with each individual having on average 17.2 segments 1.5 KB or larger. Measurements of genome-wide and regional FROH were used as the primary predictors of trait variation in association analysis.** Results:** In genome-wide FROH analysis, we did not identify any associations that withstood Bonferroni-correction (p = 0.0005). However, on regional FROH analysis, we identified associations exceeding genome-wide thresholds for two traits: serum bilirubin levels, which were significantly associated with a region on chromosome 2 localized to a region surrounding UGT1A10 (p = 1 × 10− 43), and HbA1c levels, which were significantly associated with a region on chromosome 8 localized near CHRNB3 (p = 8 × 10− 10).** Conclusions:** These analyses highlight the potential value of autozygosity mapping in founder populations.

**Supplementary Information:**

The online version contains supplementary material available at 10.1186/s12864-023-09208-5.

## Introduction

Autozygosity, which is defined as the probability that a region is homozygous due to the inheritance of alleles identical-by-descent (IBD), is determined by the presence of extended homozygosity in that region. Since runs of homozygosity (ROHs) are a recognized signature of recessive inheritance, homozygosity mapping using ROH has commonly been used to map recessive disorders when there is suspicion that parents may share a common haplotype. [1].

More recently, studies have evaluated the impact of overall autozygosity on variation in common complex traits, where overall autozygosity is estimated as the proportion of ROH across the entire genome (genome-wide autozygosity). Genetic homogeneity, estimated as overall autozygosity, has previously been linked to adverse health outcomes in multiple traits impacting evolutionary fitness. Populations with increased autozygosity are more likely to experience inbreeding depression, or reduced fitness, which has been linked to a range of phenotypic consequences including cardiovascular disease, [2, 3] shorter stature, [4, 5] lower general cognitive ability, [5] decreased fertility, [6, 7] and higher hip-to-waist ratio. [8] In case-control study designs involving outbred populations, higher genome-wide autozygosity has also been associated with coronary artery disease [3] and amyotrophic lateral sclerosis. [9] These studies raise the potential for employing ROH mapping to identify hotspots along the genome that contain multiple rare, recessive loci that influence health and disease. Autozygosity mapping may be a particularly powerful tool when applied to founder populations where consanguinity is high. [10].

Elevated autozygosity is seen in population isolates that have a small set of common founders. [11] Due to limited genetic diversity, recent inbreeding in such populations magnifies the occurrence of mildly deleterious variants, resulting in an increased burden of recessive disorders that are rare in the general population. [12] The Amish of Lancaster County, PA are a relatively recent founder population who emigrated from Europe to Lancaster 14–15 generations ago. In this study, we tested for association between degree of autozygosity and variation in 96 different complex traits among 7221 Old Order Amish individuals residing in Lancaster County, PA. We estimated genome-wide levels of autozygosity as the proportion of the autosomal genome in runs of homozygosity > 1.5 Mb. We also estimated the probability of autozygosity at 10 Kb average intervals throughout the genome. We used these measures in association analysis to assess evidence for association of genome-wide and locus-specific association of autozygosity with 96 different phenotypes.

## Methods

### Participants

Subjects in this study were recruited through multiple protocols carried out between 2003 and 2019 as part of the Amish Research Program. Study participants were recruited through the University of Maryland Amish Research Clinic (ARC) in Lancaster, PA. Studies were designed to assess determinants of cardio-metabolic and bone health in the community, and enrollment was open to volunteers throughout the Lancaster Amish community. [13, 14] Recruitment was generally phenotype agnostic; that is, subjects were not recruited for particular diseases or health conditions. This report is based on 7,221 apparently healthy Amish individuals 18 years of age or older recruited from the community and in whom we obtained genotyping data. The average age of participants was 41.8 years and the population was 43% male.

### Phenotyping

Clinical examinations were performed by trained nursing staff at the Amish Research Clinic in Lancaster, PA or in the homes of study participants. For this report, we restricted analysis to a set of phenotypes measured in common across all (or nearly all) protocols. These include basic anthropometrics, blood pressure, fasting blood lipids, glucose and insulin, hemoglobin a1c (glycated hemoglobin), basic blood chemistries, and medical histories. A complete list of the 96 phenotypes analyzed is provided in Supplemental Table 1.

### Genotyping

Genotyping was performed at the Regeneron Genetics Center using the Illumina Global Screening Array, which included 490k single nucleotide polymorphisms (SNPs) that passed quality control parameters. Samples with > 5% missingness were removed from analysis as were variants with > 2% missingness or high levels of Mendelian errors. We also applied a minor allele frequency threshold of 0.01. For these analyses we used only the unimputed genotype data.

### Genome-wide autozygosity estimation

Genome-wide autozygosity was estimated as the proportion of the genome that fell into ROHs. SNPs with more than 3% missingness across individuals and with a minor allele frequency less than 5% were excluded from ROH calculations. ROH estimates were made using the IBD tool implemented in Plink, which has several built-in arguments. [15] The key parameters to identify truly autozygous segments are minimum length (kilobase, Kb) needed for a tract to qualify as homozygous, number of contiguous homozygous SNPs, and minimum tract density requirements. To avoid very short and common strands of homozygosity that occur throughout the genome due to linkage disequilibrium (LD), we used a minimal length of ROH of 1.5 Mb. [5, 16] The fraction of each autosomal genome in ROH > 1.5 Mb correlates well with pedigree-based estimates of inbreeding. [16] All other parameters were the default parameters set by Plink except for a decrease in the number of contiguous homozygous SNPs to 50, consistent with previous studies. [5, 11] We used the PLINK default settings to allow each autozygous segment to include up to five missing SNPs and up to one heterozygous SNP. Since study individuals were genotyped with the same assay and processed under the same QC protocol, ROH was defined as the total kilobase (Kb) included in a ROH.

### Regional autozygosity estimation

Regional autozygosity computation was performed using the *GARLIC* software, which outputs ROH and length class information in UCSC’s plain-text BED format. [17] Autozygosity was estimated using a sliding window approach with a step size of 10 kb, a 0.001 genotype error rate, population-specific allele frequencies and a window size of 50 SNPs. For each individual, we extracted ROH inclusion status for each SNP on the genotyping array (i.e., SNP autozygous in the ROH segment or not) for use in the downstream association analysis. Each SNP was assigned a value of 1 indicating that the SNP is included in an ROH or a value of 0 indicating that the SNP is not included in an ROH. Therefore, each SNP now represents the status of regional autozygosity.

### Statistical analysis

We tested for association of the autozygosity estimates (genome-wide and regional) with trait variation using a linear mixed model with phenotype as the outcome and autozygosity as the independent variable. As fixed covariates for each trait analysis, we included age, sex, and genotypes for two large effect SNPs (*APOB* p.Arg3527Gln and *APOC3* p.Arg19Ter) previously identified in this Amish community affecting lipid levels. [18, 19] We also included a genomic relationship matrix (GRM) as a random effect to account for covariation among related individuals. [20] Genome wide or regional F_ROH_ was used as the primary predictor of the traits of interest. The genome wide significance threshold after Bonferroni correction was p = 0.0005 (0.05/96 traits). As a secondary analysis, we tested for sex-specific effects of autozygosity on trait variation using an autozygosity*sex interaction term. For regional analysis, an independent association analysis was performed for each of 300k SNPs using ROH status as the primary predictor of the 96 traits of interest. We adjusted the significance threshold to account for 170k independent SNPs among these 300k using an LD threshold of r^2^ = 0.50. The Bonferroni adjusted significance threshold was p = 3.1 × 10^− 9^.

## Results

### ROH across the genome

**Fig. 1:**
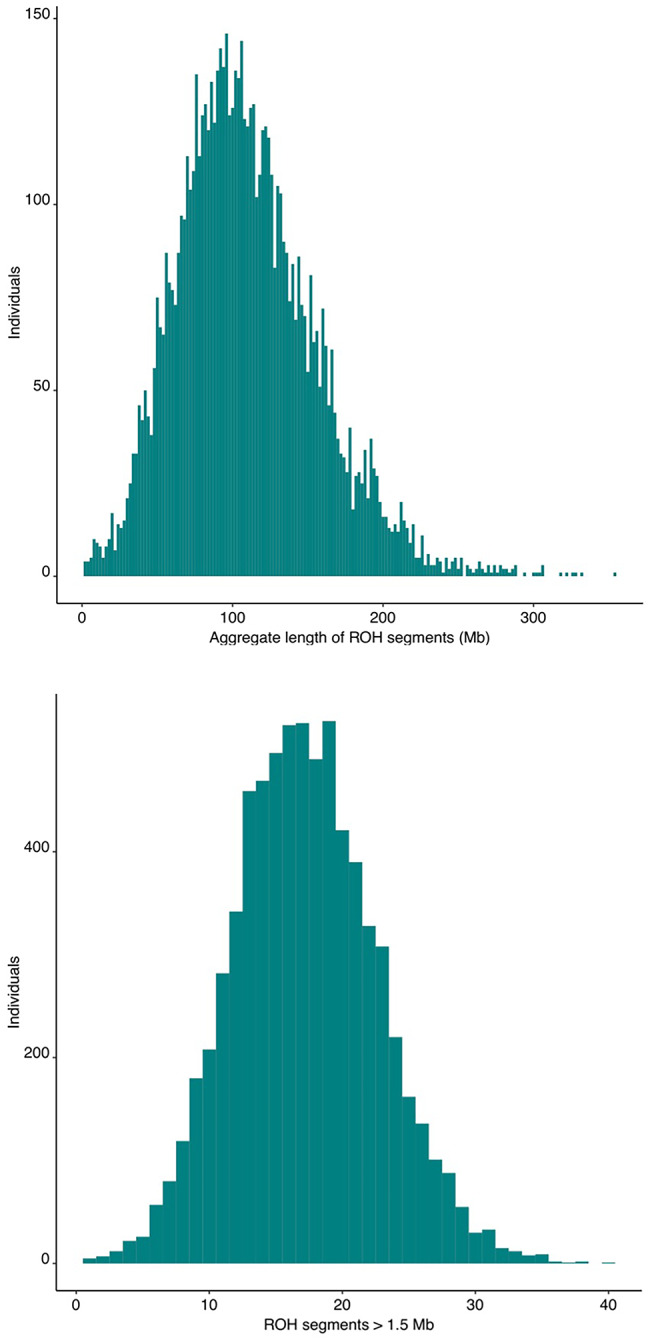
Histograms of ROH segment length and number. Participant had an average of 17.2 segments of > 1.5 Mb, which spanned an average of 110 Mb a) Frequency distribution of the total length of ROH segments across the genome (Mb) per subject b) Frequency distribution of the total number of ROH segments present along the genome per subject

On average, each participant had 17.2 segments of > 1.5 Mb, which in aggregate spanned 110 Mb, or ~ 3.7% of the genome (Fig. 1). ROH were present on each autosome and were widely distributed across the genome. We assessed the genome-wide distribution of ROHs by the frequency of entire ROH segments and by the frequency in which SNPs are included in an ROH segment.

**Fig. 2:**
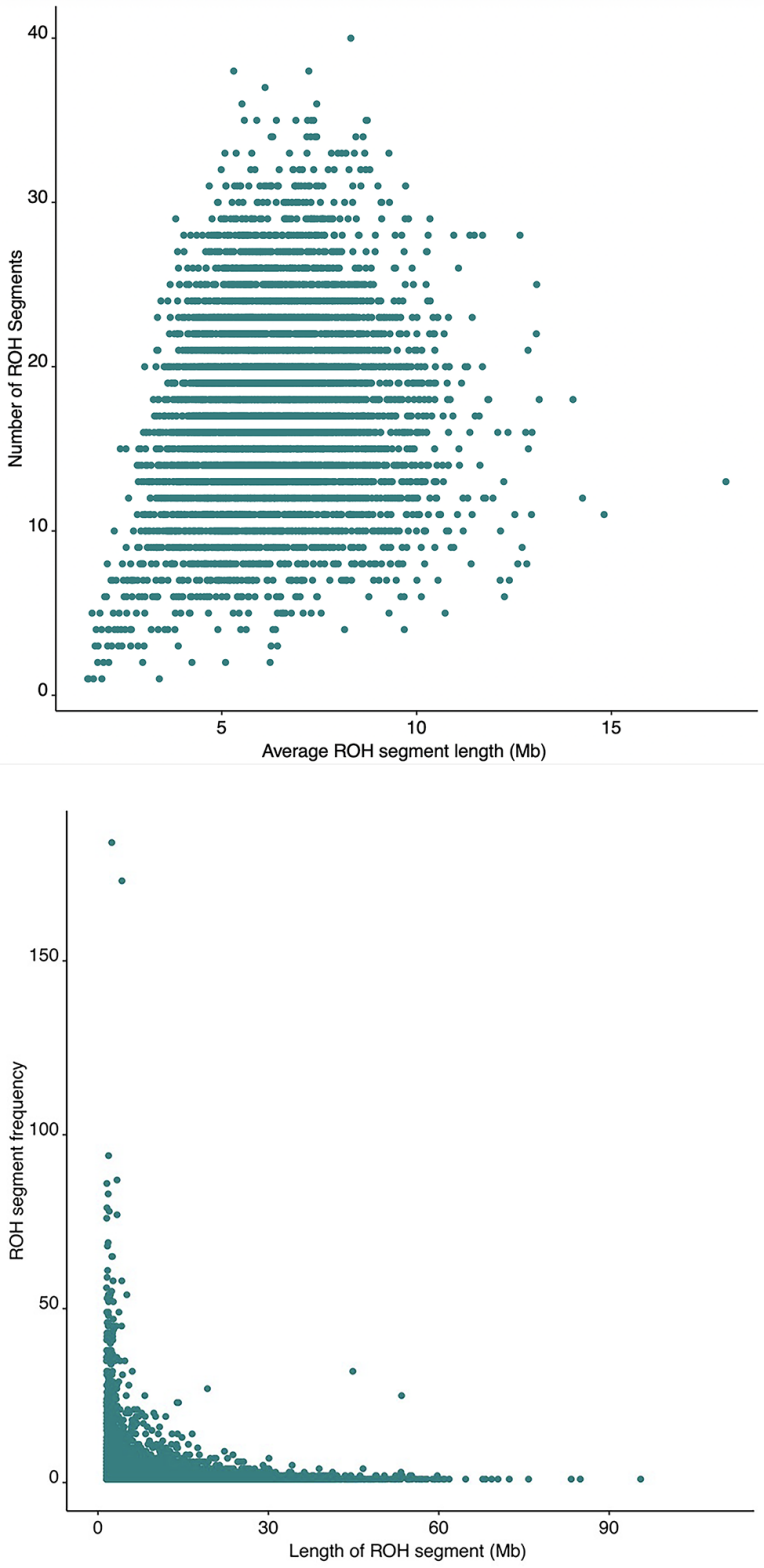
Size distribution of ROH segments a) Plot showing the total number and average length of autozygous segments. Each dot corresponds to one individual b) Plot showing the frequency and length of autozygous segments. Each dot corresponds to one autozygous segment identified within at least one member of the study population

In general, shorter ROH segments occurred more frequently than longer ROH segments, with a mean ROH segment length of 6.3 Mb (Fig. 2A and B). Two short segments had notably high frequencies (Fig. 2B). The highest frequency segment is located at chr1: [145,927,328: 148,353,534] and is 2.4 Mb long, occurring in 184 individuals. The segment with the second highest frequency, occurring in 173 individuals, is located at chr10: [42,113,412: 46,332,633], and is 4.2 Mb long.

**Fig. 3:**
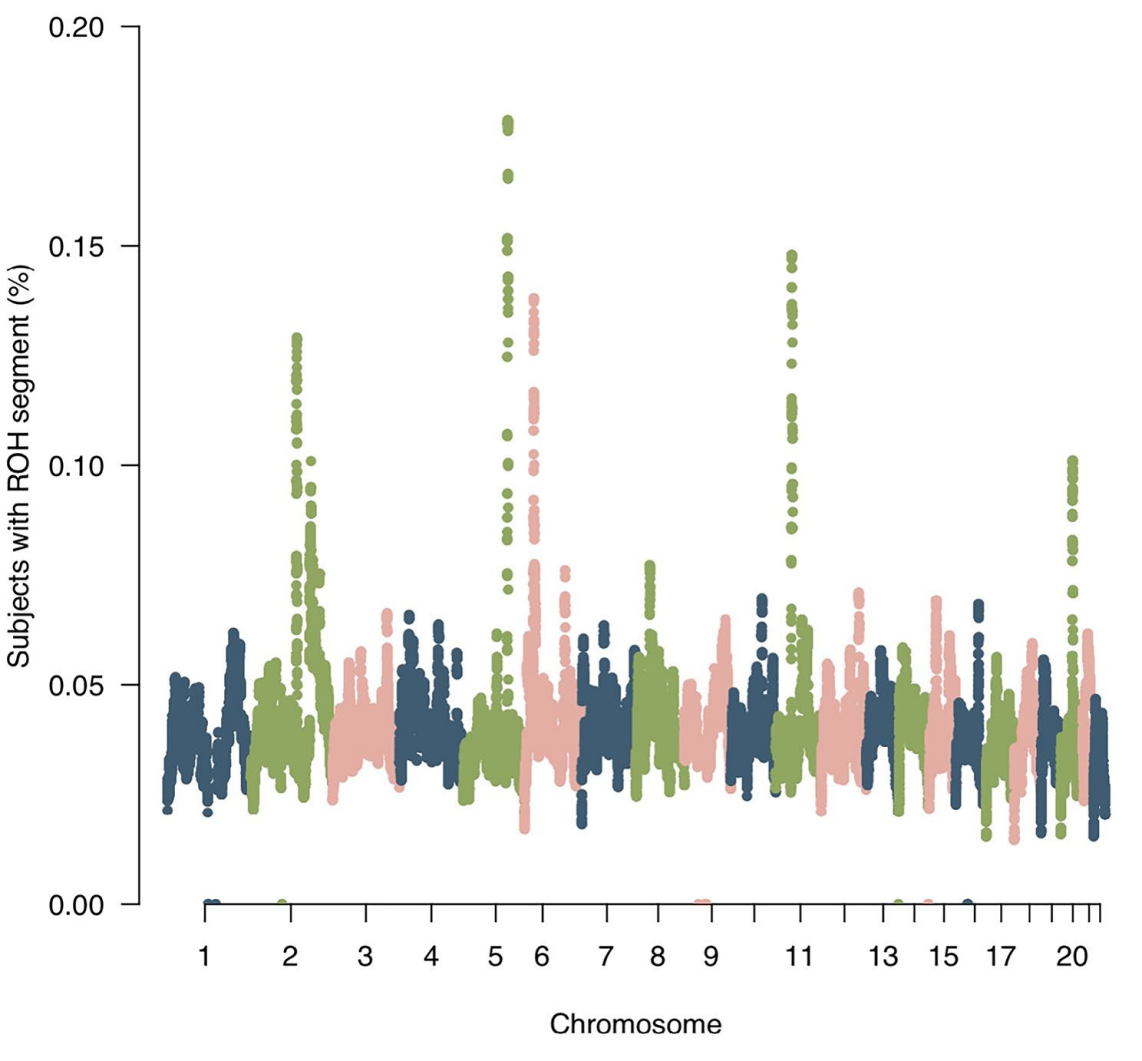
Frequency of ROH segments across the autosome. Manhattan plot showing the frequency that a SNP occurred within an autozygous segment in the cohort

There are several regions of the genome for which the frequency of individuals with SNPs in ROH segments was particularly high (chr 2: [134600135:136231566]; chr 2: [176,901,247: 178,324,285]; chr 5: [130334251:132873169]; chr 6: [27,512,529: 29,306,571]; chr 11: [47253513:49758165]; chr 20: [34813904:35819776]) (Fig. 3, Supplemental Table 2). For example, 18% of individuals had ROH in a region on chromosome 5 containing genes *RAPGEF6*, *FNIP1*, and *ACSL6*. This region has previously been identified as having enriched ROH frequency for SNPs. [21] In chromosome 2, 13% of subjects had ROH in a region that includes *LCT*, known to have recent positive selection in European ancestry individuals, [22] and genes *UBXN4* and *R3HDM1*, which have not previously been noted in ROH studies. Our finding that a relatively high proportion of subjects (14%) had ROH on chromosome 11 in a region harboring *FOLH1* and *OR4A47* is consistent with previous findings. [21].

Almost all SNPs fell into an ROH at least once, except in six instances across the genome in which small clusters of SNPs did not belong in any ROH segment (Fig. 3, Supplemental Table 3). This may indicate that diversity is favored and ROHs are not well tolerated in these regions.

### Association of genome-wide ROH with health and disease-related traits

Measurements of F_ROH_ were then used as the primary predictor of phenotypic variation. We analyzed 96 traits including basic anthropometrics, blood pressure, fasting blood lipids, glucose, insulin, HbA1c, basic medical blood chemistry measurements, and medical histories. We did not identify any associations that withstood Bonferroni-correction (p < 0.0005), but the lead association (p = 0.0036, b = 2.5 × 10^− 5^, se = 8.3 × 10^− 6^) with genome-wide F_ROH_ was with electrocardiogram (EKG) QT interval, followed by serum levels of CO_2_ (p = 0.03, beta = 1.5 × 10^− 6^, se = 7.3 × 10^− 7^), urea nitrogen (p = 0.04, beta = 2.5 × 10^− 6^, se = 1.2 × 10^− 6^), and thyroid hormone measurement (tsh) (p = 0.04, beta = 4.6 × 10^− 6^, se = 2.3 × 10^− 6^). In a sex-stratified analysis, we did not identify any sex-specific effects of F_ROH_.

### Association of regional autozygosity with health and disease-related traits

**Fig. 4:**
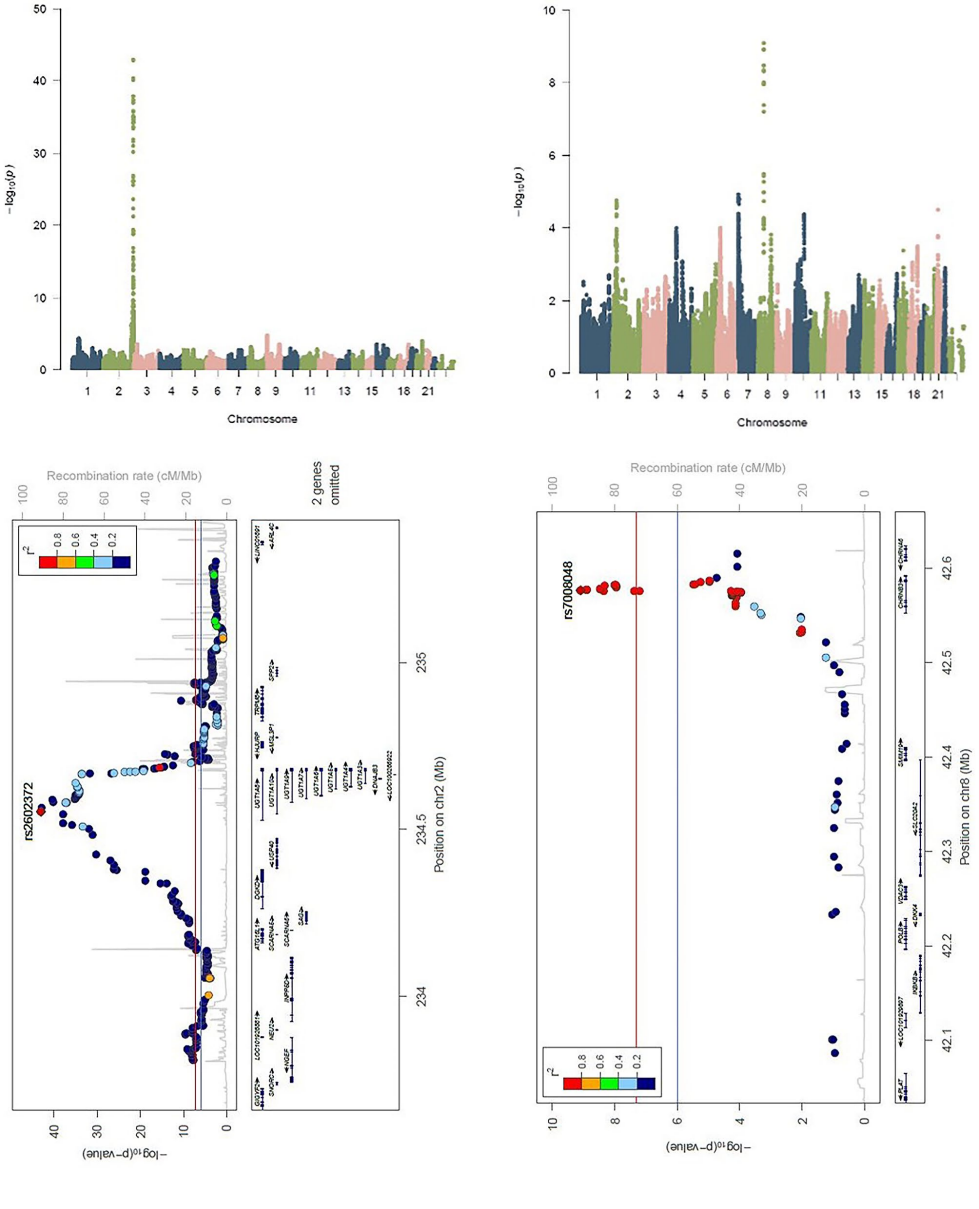
Autozygosity mapping Manhattan and zoom plots Manhattan plot of (a) serum bilirubin levels and (b) HbA1c levels. Each dot represents a SNP. For statistical analysis, we extracted ROH inclusion for each SNP on the genotyping array Zoom plot of F_ROH_ and c) serum bilirubin levels and d) HbA1c levels. Red line indicates the significance threshold of P = 3.1 × 10^− 9^

We analyzed 96 traits for associations with regional autozygosity levels and identified four trait associations at genome-wide significance following Bonferroni correction (p = 3.1 × 10^− 9^) (Fig. 4a-d). Increased serum bilirubin levels were significantly associated (p = 1 × 10^− 43^, beta = 0.19, se = 0.013) with increased F_ROH_ at a region of chromosome two that includes the *UGT1A10* gene. As we [23] and others [24] have previously reported an association of a nearby SNP in *UGT1A1*, a gene encoding an enzyme that converts bilirubin into a more water-soluble form that the body is better able to excrete, we adjusted for the SNP as an additional fixed effect but found the ROH association to be essentially unchanged (beta = 0.19, p = 1.1 × 10^− 43^ vs. beta = 0.19, p = 1.7 × 10^− 44^). Three additional traits were associated with increased F_ROH_, HbA1c (p = 8 × 10^− 10^, beta = 0.17, se = 0.027) with a region on chromosome eight surrounding the *CHRNB3* gene, C-reactive protein (p = 2.7 × 10^− 9^, beta = 3.17, se = 0.53) with the intergenic region of *FBXO33* and thyroid hormone levels (p = 2.8 × 10^− 9^, beta = 3.14, se = 0.53) with the intergenic region of *LRRC3B* (Supplemental Fig. 1a-b). To our knowledge, no genes previously linked to these traits are close to these signals.

### Recessive based SNP analysis

To test whether the regions associated with bilirubin and HbA1c identified through ROH analysis would be detected with other methods, we set up a recessive mode association analysis. We detected a strong association (lowest p value = 1 × 10^− 464^) between Bilirubin levels and SNPs within the same region that was detected with ROH analysis. HbA1c levels were not strongly associated (p > 9.6 × 10^− 4^) with any SNPs in the region when assessing using a recessive model.

## Discussion

### ROH across the genome

The average Amish individual had autozygous segments spanning ~ 3.7% of their genome. In comparison, individuals with European ancestry from the UK biobank had ~ 0.4%.^11^ When assessing ROHs with a cutoff of > 1 Mb, Amish participants harbored 18.6 ROH segments on average with an average length of 5949 Kb. In contrast, non-founder European populations harbored 8.02 segments on average with an average length of 1421 Kb. [25] We were not able to compare distributions of ROHs in Amish with UK biobank because subject level data was not available. However, the observation that ROHs are longer and more numerous in this founder population compared to outbred populations is not surprising given that longer shared haplotypes are inherited from recent common ancestors and reduced effective population size, due to a population bottlenecks, increases the number of ROH present. [26].

ROHs were widely distributed across the genome and were present on each autosome. The region with the highest frequency of SNPs in an ROH was identified in chromosome 5. The *LCT* gene in chromosome 2 had an enrichment of frequency of ROH which is consistent with previous studies. [22] Although an enrichment was also seen on chromosome 6, it was not localized to the MHC locus as seen previously. [25, 27] Across the genome, there were just six instances in which small clusters of SNPs were not included in any ROH segments. Five of these regions contain pseudogenes and noncoding RNA (Supplemental Table 3). This may indicate that diversity is well tolerated, and the regions are not under selective pressure. The sixth instance is along chromosome 14, in a region containing the gene *OR11H12*. This gene is a member of the olfactory receptor proteins and is known to have copy number variations. [28].

### Association of genome-wide ROH with traits of interest

We have tested for associations between autozygosity, as measured by ROH, and a large panel of complex traits in a founder population of Amish individuals. We did not identify associations of genome-wide autozygosity with any traits that withstood Bonferroni-correction. In contrast, a large meta-analysis of 234 cohorts tested the association of genome-wide autozygosity levels with 100 traits and found significant associations with 6 cardiometabolic traits that were included in our analysis. [11] These cohorts were mostly non-founder but included the Lancaster Amish cohort from the present study, Hutterites, and several others with high consanguinity.

In the Amish, the lead association was between higher autozygosity measurements and QTc interval. Because the Amish study population is enriched for a known, high effect size, *KCNQ1* pathogenic variant, p.Thr244Met, that increases QTc intervals, [29] we removed the *KCNQ1* genomic region from the analysis and re-assessed the association. The association, although not statistically significant after Bonferroni correction, was still present and did not diminish.

### Association of regional autozygosity with traits of interest

Previously, ROH mapping has been used to detect loci with a segregating recessive variant. This method is regarded as more powerful than traditional single-marker association studies using a recessive model because there is more certainty of the haplotype on which the two alleles appear and greater viability at extreme minor allele frequencies. [9]*GARLIC* and other ROH prediction tools have been used for case-control studies and whole genome F_ROH_ associations with complex phenotypes. Here, we implemented *GARLIC* to map regional associations for complex trait discovery.

We detected associations of increased F_ROH_ with higher levels of serum bilirubin levels at a region on chromosome 2 with increased HbA1c at a region on chromosome 8. The mapping analysis also identified an association with cholesterol clustering around the *APOB* region on chromosome 2 which contains a high effect size variant p.Arg3527Gln. [18] This association was between increased LDL-C levels and decreased autozygosity. Therefore, heterozygotes in this region are driving the association.

### Strengths and limitations

Population isolates have an increased burden of ROH and can uncover genomic regions associated with complex phenotypes that may not have been identified in populations with more distant parental relatedness. Due to low levels of genome-wide homozygosity commonly seen in modern human populations, very large numbers of study subjects are required to provide sufficient statistical power. [16] Although no complex traits in our study were associated with genome-wide autozygosity levels, this could potentially be due to limited power given the sample size or because many examined phenotypes were cardio-metabolic which are risk factors for late-onset conditions and may not be under evolutionary selective pressure. [5] Also, genome-wide autozygosity measurements may not predict some polygenic traits influenced by dominant alleles at different loci, each influencing the trait in opposite directions. To identify genomic regions with dominant or additive alleles driving trait associations, GWAS are a better method than regional autozygosity mapping. For example, GWAS identified a statistically significant association between *UGT1A10* and bilirubin levels (p = 2 × 10^− 38^ for *UGT1A10* rs17854828 in 5830 subjects), although an association between HbA1c levels and chromosome 8 has not previously been reported.

The lead SNP from the recessive model may be too common (MAF = 41.7%) to exist on just one haplotype. Therefore, the signal may be diluted when using the ROH method. The causal SNP is found in a large fraction of the population but is not always present on the haplotype with the strongest effect. The SNPs in the region associated with HbA1c levels are rare and are only found in individuals that share a common haplotype.

## Summary

Amish have larger ROH segments and more of the Amish genome included in autozygous regions compared to outbred populations. Genome-wide summed autozygosity was not significantly associated with any of the 96 traits tested in this study. Using regional autozygosity mapping methods, we identified two traits associated with regional levels of autozygosity. We found that increased serum bilirubin levels were associated with increased autozygosity on chromosome two, localized to the *UGT1A10* gene and that increased HbA1c levels were associated with increased autozygosity on chromosome eight, localized to the *CHRNB3* gene.

## Electronic supplementary material

Below is the link to the electronic supplementary material.

**Additional file 1: Supplemental Figure 1.** Zoom plot showing associations between FROH and a) C-reactive protein and b) thyroid hormone. Red line indicates the significance threshold of P = 3.1 x 10-9.

**Additional file 2:** **Supplemental Table 1.** "Panel of complex traits measured in Amish participants that were tested for association with regional and global autozygosity." - this title is incorrect in the current proof.

**Additional file 3: Supplemental Table 2.** "SNPs with the highest frequency of inclusion in an ROH (frequency = 0.09 – 0.18). The SNPs that are shown were found on chromosome 2, 5, 6, 11, and 20."

**Additional file 4:** **Supplemental Table 3.** "SNPs with the lowest frequency of inclusion in an ROH (frequency = 0.00 – 0.015)."

**Additional file 5.**

## Data Availability

The datasets used and/or analysed during the current study available from the corresponding author on reasonable request. Some deidentified Amish data are available on dbGaP (dbGaP Study Accession: phs000956.v3. p1). Further inquiries may be directed to the communicating author.
