# Supplementary figures and images for: Associations of genome-wide and regional autozygosity with 96 complex traits in old order Amish
Source: BMC Genomics. 2023 Mar 20;24:134. doi: 10.1186/s12864-023-09208-5 (PMC10029202; doi:10.1186/s12864-023-09208-5)

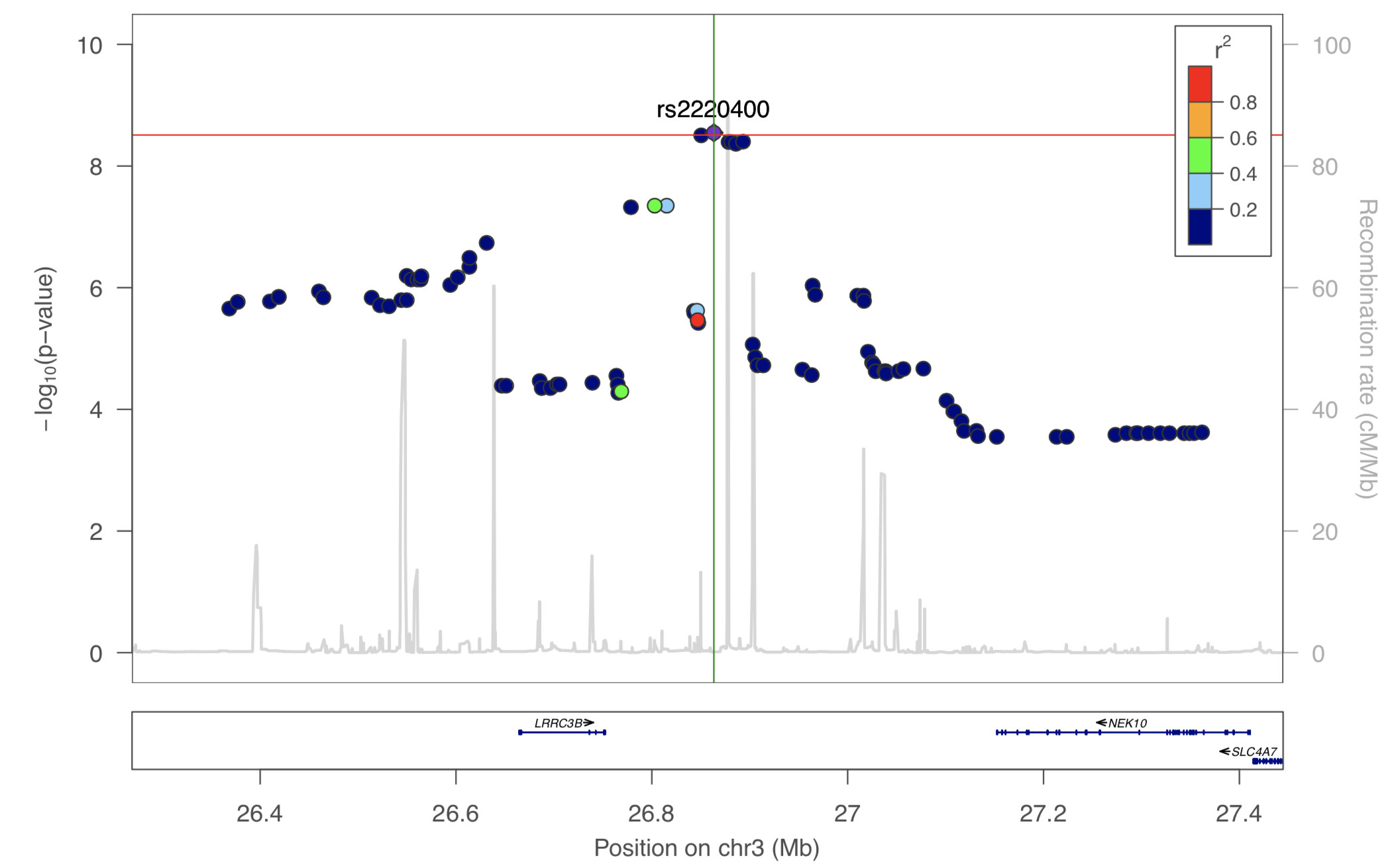

Supplement: Supplementary file 3 — Additional file 3: Supplemental Table 2. "SNPs with the highest frequency of inclusion in an ROH (frequency = 0.09 – 0.18). The SNPs that are shown were found on chromosome 2, 5, 6, 11, and 20." [file 12864_2023_9208_MOESM3_ESM.jpg]

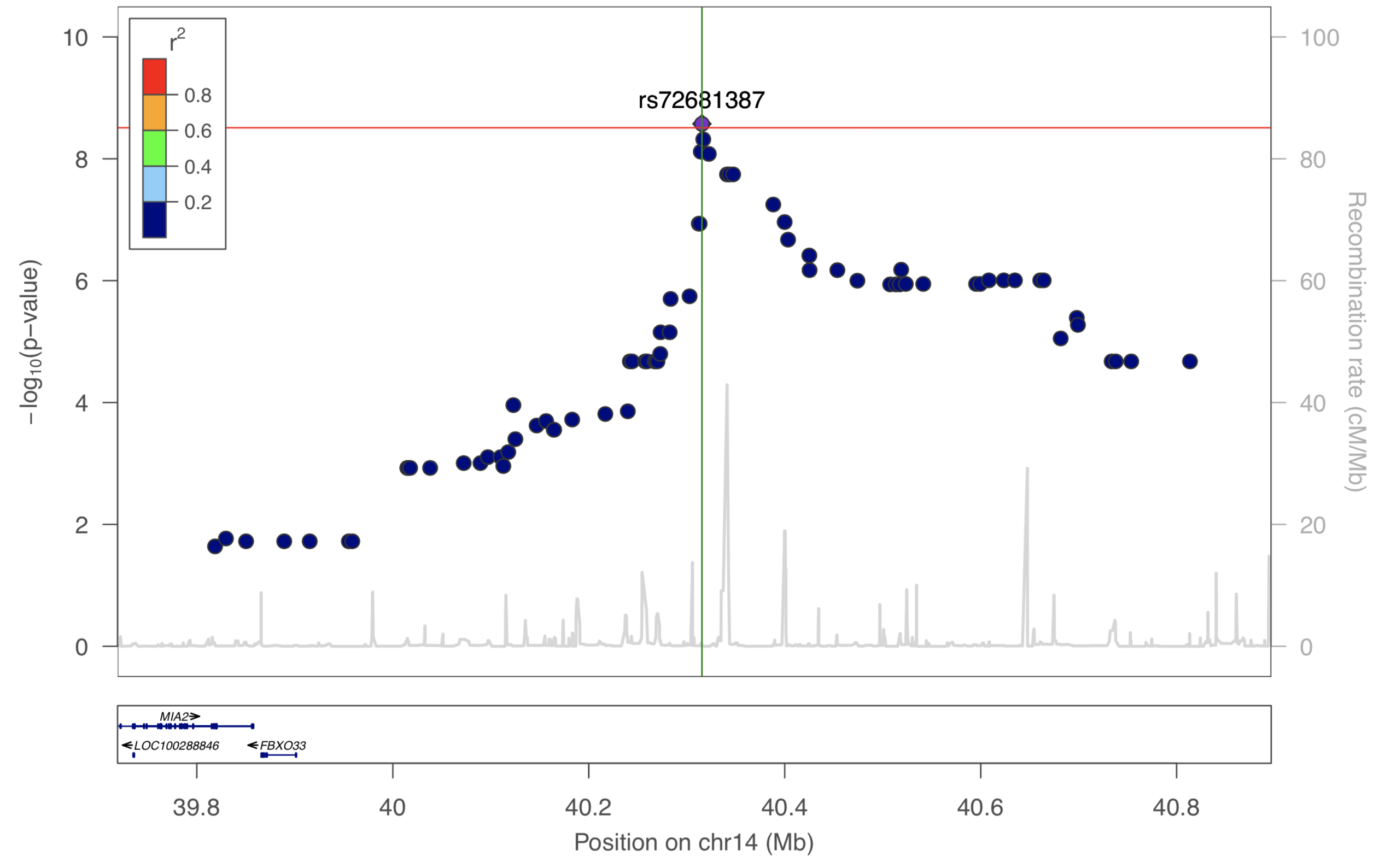

Supplement: Supplementary file 4 — Additional file 4: Supplemental Table 3. "SNPs with the lowest frequency of inclusion in an ROH (frequency = 0.00 – 0.015)." [file 12864_2023_9208_MOESM4_ESM.jpg]
