# Supplementary material for: Associations of genome-wide and regional autozygosity with 96 complex traits in old order Amish
Source: BMC Genomics. 2023 Mar 20;24:134. doi: 10.1186/s12864-023-09208-5 (PMC10029202; doi:10.1186/s12864-023-09208-5)
Supplement: Supplementary file 5 — Additional file 5. [file 12864_2023_9208_MOESM5_ESM.docx]

Supplemental Figure 1. Zoom plot showing associations between FROH and a) C-reactive protein and b) thyroid hormone. Red line indicates the significance threshold of P = 3.1 x 10-9.
